# Phase IB study of Avelumab and whole brain radiotherapy in patients with leptomeningeal disease from solid tumors: Results and molecular analyses

**DOI:** 10.1093/neuonc/noaf183

**Published:** 2025-08-28

**Authors:** Yolanda Piña, Vincent Law, Solmaz Sahebjam, Nam Tran, Navya Siddarajappa, Jiannong Li, Qianxing Mo, Manali S Phadke, John Arrington, Robert Macaulay, Sepideh Mokhtari, Brittany Evernden, Kamran A Ahmed, Inna Smalley, Michael Yu, Keiran S M Smalley, Peter A Forsyth

**Affiliations:** Department of Neuro-Oncology, H. Lee Moffitt Cancer Center, Tampa, Florida, USA; Department of Neuro-Oncology, H. Lee Moffitt Cancer Center, Tampa, Florida, USA; Department of Neuro-Oncology, H. Lee Moffitt Cancer Center, Tampa, Florida, USA; Department of Neuro-Oncology, H. Lee Moffitt Cancer Center, Tampa, Florida, USA; Department of Tumor Microenvironment and Metastasis, H. Lee Moffitt Cancer Center, Tampa, Florida, USA; Department of Biostatistics and Bioinformatics, H. Lee Moffitt Cancer Center, Tampa, Florida, USA; Department of Biostatistics and Bioinformatics, H. Lee Moffitt Cancer Center, Tampa, Florida, USA; Department of Tumor Microenvironment and Metastasis, H. Lee Moffitt Cancer Center, Tampa, Florida, USA; Department of Radiology, H. Lee Moffitt Cancer Center, Tampa, Florida, USA; Department of Neuropathology, H. Lee Moffitt Cancer Center, Tampa, Florida, USA; Department of Neuro-Oncology, H. Lee Moffitt Cancer Center, Tampa, Florida, USA; Department of Neuro-Oncology, H. Lee Moffitt Cancer Center, Tampa, Florida, USA; Department of Radiation-Oncology, H. Lee Moffitt Cancer Center, Tampa, Florida, USA; Department of Metabolism and Physiology, H. Lee Moffitt Cancer Center, Tampa, Florida, USA; Department of Radiation-Oncology, H. Lee Moffitt Cancer Center, Tampa, Florida, USA; Department of Tumor Microenvironment and Metastasis, H. Lee Moffitt Cancer Center, Tampa, Florida, USA

**Keywords:** Avelumab, immunotherapy, leptomeningeal disease, radiotherapy

## Abstract

**Background:**

Leptomeningeal disease (LMD) from solid tumors has a dismal prognosis, even following treatment with anti-PD-1 therapy. We performed a phase IB study evaluating the safety of Avelumab with whole brain radiotherapy (WBRT) in LMD (NCT03719768).

**Methods:**

Fifteen patients were enrolled with LMD from breast, lung, nasopharyngeal, ovarian, and pancreatic tumors. Patients were treated with Avelumab with WBRT, with the first infusion of Avelumab starting 14 days pre-WBRT and continuing during and post-WBRT for up to 5 cycles. Primary endpoints were safety and 3-month OS (OS3). Secondary endpoints included assessment of immune cells in the cerebrospinal fluid (CSF) using single-cell RNA-sequencing (scRNA-Seq) pre- and post-last treatment of Avelumab.

**Results:**

DLTs occurred in 2 patients, ie, adrenal insufficiency, hypothyroidism, and pneumonitis. Treatment-related toxicities occurred in 5 patients with grade 1/2 and 5 patients with grade 3/4. Immune-related adverse events occurred in 5 patients with grade 1/2 and 3 patients with grade 3/4. The OS3 was 67% (10 of the 15; 95% CI: 38%–84%). Median-OS was 3.85 months (95% CI: 0.9–34.4 months) and median-PFS was 3.85 months (95% CI: 0.9–12.1 months). scRNA-Seq analysis of CSF pre- and post-last-treatment showed Avelumab + WBRT stimulated an adaptive immune response associated with a decrease in regulatory T cells (Tregs), among other changes in the expression of immune checkpoints on CD8 + T cells and macrophages.

**Conclusions:**

The combination of Avelumab and WBRT is safe and demonstrates activity in patients with LMD. The identification of high levels of Tregs and macrophages in the CSF of LMD patients offers future avenues for therapeutic development.

**Importance of the Study**

Patients with LMD have a grave prognosis with a median survival measured in weeks to a few months. Once thought to be a “rare” disease, the incidence of LMD is increasing rapidly due to improved survival associated with new systemic therapies. Only a handful of prospective trials have been completed in LMD to date, and little is known about the mechanisms of response and resistance within the CSF space. In this study, we undertook a phase 1B trial to evaluate the safety of Avelumab + WBRT in LMD, along with high-dimensional analysis of pre-and post-treatment CSF samples using scRNA-Seq. We demonstrated that Avelumab + WBRT is safe, with signs of clinical activity. Correlative studies showed treatment associated with a decrease in Tregs and changes in the activation of CD8 + T cells and macrophages. Our findings serve as a foundation for the development of future phase II trials with Avelumab + WBRT, along with more focused correlative studies, in LMD.

**Key Points**

- Avelumab + WBRT was safe and well-tolerated in the cohort of patients evaluated.
- Correlative studies showed Avelumab + WBRT to stimulate an adaptive immune response in the CSF space.
- Results were encouraging and serve as a foundation for a Phase 2 trial in LMD.

Leptomeningeal disease (LMD) is a devastating complication from cancer, most commonly seen in breast (2%–5%), lung (3%–10%), and melanoma (5%–25%).^1–5^ Despite advances in targeted and immunotherapies, LMD has a poor median overall survival (OS) of 8–10 weeks and modest improved survivals with treatment of 3.3–4.5 months with chemotherapy,^2,6,7^ 4.9–6.3 months with immunotherapy,^8,9^ ~2 months with photon craniospinal irradiation (CSI) alone,^10,11^ ~4–8.8 months with combination of photon whole brain radiation (WBRT) +/− spine radiation or CSI and chemotherapy,^10,12,13^ and 3–9.9 months with proton CSI (pCSI).^11,14,15^ LMD can involve any part of the central nervous system (CNS) and cause any variety of neurological signs and symptoms. LMD remains a diagnostic challenge with the “Gold Standard” of positive cerebrospinal fluid (CSF) cytology being falsely negative in up to 50% of cases, and the diagnosis relies, oftentimes, on radiographic and clinical findings.^16^

Although the development of immune-checkpoint inhibitors (ICIs) revolutionized treatment for melanoma, lung, and breast cancers, their effectiveness in patients with LMD is unclear. Avelumab exerts its effects through enhanced cytotoxic T-cell responses by blocking the interaction between PD-L1 and PD-1 and B7-H1, and is currently FDA-approved for the treatment of metastatic Merkel cell carcinoma, urothelial carcinoma, and renal cell carcinoma.^17–22^ The antitumor activity of Avelumab has been well studied in preclinical models and clinical trials,^23–26^ and is being investigated in multiple human cancers. The JAVELIN clinical trial using Avelumab in metastatic urothelial cancer showed it to be safe with a good overall response rate (RR) of 16.1% at >6 months of follow-up.^27^

As anti-PD-1 therapy has been disappointing in LMD as monotherapy, we explored whether the combination of PD-1/PD-L1 blockade with Avelumab in combination with WBRT would be more effective. There is preclinical evidence that the best tumor control with the highest tumor-specific T-cell response and lowest regulatory T-cell counts was achieved with higher radiation therapy (RT) dose per fraction, and that fractionated RT can induce an abscopal effect when combined with immune checkpoint inhibition therapy in preclinical carcinoma models.^28,29^ WBRT (3000 cGy ×10 fractions) was considered to be the most appropriate dosage to balance long-term toxicity with an enhanced immune response. We hypothesized that (1) systemic Avelumab + WBRT increases activated T cells in the CSF, (2) is safe without significant toxicities and improved OS, and (3) point estimates (with confidence intervals [CIs]) of CSF and clinical parameters can be used to estimate sample sizes for an expanded Phase II trial. Therefore, in this open-label phase IB trial, we tested the safety and efficacy of Avelumab + WBRT in LMD (NCT03719768).

## Methods

### Study Design and Patients

We conducted a Phase 1B study of Avelumab + WBRT in patients with LMD from different primary solid cancers (NCT03719768). Eligible patients had histologically or cytologically confirmed diagnosis of a solid tumor, positive CSF cytology and/or clinical signs and symptoms of LMD with characteristic radiographic abnormalities on MRI,^16,30^ Karnofsky Performance Status of 50 or higher, interval of at least 2 weeks after the end of prior RT to the brain, interval of at least 4 weeks following any brain surgical resection prior to treatment, and be 18 years of age or older on the day of signing consent. Patients who had prior PD-1/PD-L1/CTLA-4/CD137 targeting therapy within 6 months prior to study treatment were excluded. Also, patients who had an active autoimmune disease requiring systemic treatment within the past 3 months (ie, with the use of disease-modifying agents, corticosteroids, or immunosuppressive agents) or had a diagnosis of immunodeficiency were excluded. Patients with vitiligo or resolved childhood asthma/atopy were an exception to this rule. Subjects that required intermittent use of bronchodilators or local steroid injections were not excluded from the study. Subjects with hypothyroidism stable on hormone replacement or Sjogren’s syndrome were not excluded from the study. Replacement therapy (eg, thyroxine, insulin, or physiologic corticosteroid replacement therapy for adrenal or pituitary insufficiency, etc.) was not considered a form of systemic treatment. Subjects with a known additional malignancy that was progressing or required active treatment were excluded, except for basal cell carcinoma of the skin, squamous cell carcinoma of the skin, or in situ cervical cancer that underwent potentially curative therapy. Patients with an active and noninfectious pneumonitis, active infection requiring systemic therapy, or major surgery in the past 28 days prior to signing consent were excluded.

If patients had disease progression while on treatment in the CNS before consent, patients were allowed to continue Her 2 directed antibody treatment (trastuzumab and pertuzumab), aromatase inhibitor, or tamoxifen while on the study; patients with triple negative breast cancer were allowed to continue capecitabine, eribulin, or paclitaxel while on the study per PI discretion.

A 30% dose-limiting toxicity (DLT) rate was considered acceptable in this study. To protect patient safety, DLTs were continuously monitored using a Pocock-type boundary,^31^ which yielded the probability of crossing the boundary at most 25% at a one-sided alpha of 13% when the rate of DLTs was 30%.

The study protocol was approved by the institutional review board. The study was conducted in accordance with the Declaration of Helsinki and Good Clinical Practice, as defined by the International Conference on Harmonization. All patients provided written informed consent prior to enrollment.

### Study Procedures

Patients received Avelumab 800 mg intravenous (IV) every 2 weeks (as a fixed dose) concurrent with WBRT 3000 cGy in 10 daily fractions, with the first infusion of Avelumab starting 14 days (day 1) prior to initiating WBRT, and continued during WBRT (days 15 and 29) and post-WBRT for up to 5 cycles (until disease progression or unacceptable toxicity). Each cycle was equivalent to 4 infusion treatments with Avelumab.

CNS response assessments were performed by the investigator using contrast-enhanced magnetic resonance imaging (MRI) at baseline, and every 2–3 months using the proposal criteria from the RANO Leptomeningeal Metastasis Group to assess response to treatment.^32^

### Outcomes

The primary endpoints were safety and the proportion of patients surviving at least 3 months after the first dose of Avelumab (OS3). The secondary endpoints were: (1) the immunocellular profiles of the CSF measured before and after treatment, (2) LMD/CNS RR; (3) OS and progression-free survival (PFS).

A DLT was defined as any one of the following adverse events (AEs) occurring within 28 days from the first dose of Avelumab. CNS toxicities were any grade (gr.) 3 or higher CNS AEs, including but not limited to cerebral hemorrhage and new-onset neurologic deficit. Non-CNS toxicities were any gr. 3 or higher nonhematologic AE except for alopecia and fatigue; gr. > 3 nausea, vomiting, or diarrhea despite maximal medical therapy; gr. > 3 laboratory values if medical intervention was required to treat the patient, or the abnormality led to hospitalization; and any gr. 3 or 4 events that did not improve within 6 weeks.

### Statistical Analysis

Categorical variables such as patient demographics, baseline characteristics, safety, and toxicities were summarized descriptively using frequencies and percentages. The 95% confidence interval (CI) of the binary variable was calculated using the Clopper-Pearson method. OS was defined as the time from the on-study date to the date of death from any cause, with censoring at the last date the patients were known to be alive. PFS was defined as the time from the on-study date to the date of documented disease progression or death, whichever occurred first; the patients were censored at the last date known to be alive without progression. Patient OS and PFS were estimated using Kaplan–Meier method. The 95% CI for CNS RR was calculated using Wilson’s method. Statistical analyses were performed using R software version 4.2.0 (https://www.R-project.org) and SAS® software version 9.4 (SAS Institute Inc., Cary, NC, USA).

### Single Cell RNA Sequencing Analysis

CSF and blood were collected before treatment and at different time points after treatment was initiated in all patients. All the CSF samples were obtained from the Ommaya reservoirs, to prevent further variability related to anatomical location, and following our standard institutional protocol for CSF collection. A limited number of samples collected were evaluated with single-cell RNA sequencing (scRNA-seq) due to a lack of funding. A total of 9 specimens were analyzed from 5 patients (4 of these matched pairs) with breast cancer LMD. CSF specimens were immediately placed on ice and transferred for processing. Samples were enriched for live cells using FACS sorting prior to quantification and loading. A single-cell suspension from each sample was quantified and analyzed for viability using the Nexcelom Cellometer K2 and then loaded onto the 10× Genomics Chromium Single Cell Controller for scRNA-seq library preparation (10× Genomics, Pleasanton, CA). The cDNA was amplified and purified, and cDNA libraries were then prepared in bulk reactions using the 10X Chromium Single Cell 3′ Library Prep Kit. Approximately 25 000 to 50 000 mean sequencing reads per cell were generated on the Illumina NextSeq 500 instrument using v2.5 flow cells. Demultiplexing, barcode processing, alignment and gene counting were performed using the 10× Genomics CellRanger software (SCIGA, RRID:SCR_021002). A 2-component computational tool, ISCVA, Interactive Single Cell Visual Analytics,^33^ was used to perform scRNA-seq analyses and visualization. ISCVA consists of 2 major components: the back-end scripts, functions utilizing R packages (eg, Seurat^34^ and SingleR^35^) for quality control and cell type classification, and the front-end JavaScripts for interactive investigation. The web tool (along with all of the data analyzed herein) can be accessed at http://iscva.moffitt.org. Cell–cell interactions were analyzed using CellChat.^36^ The full dataset is also available at GEO (accession number GSE299082).

## Results

### Patients

From June 2019 to April 2022, a total of 16 patients were consented and enrolled in the study. Of these patients, 1 patient did not receive study therapy owing to screening laboratory work-up, making the patient ineligible. The analytic cohort consisted of 15 patients who were enrolled and received Avelumab and WBRT. One patient did not complete WBRT secondary to LMD disease progression.

Disease sites included breast (*n* = 8), non-small cell lung cancer (NSCLC; *n* = 4), undifferentiated sinus tumor (*n* = 1), ovary (*n* = 1), and pancreas (*n* = 1; **Table 1**). The breast cancer cohort included one patient with human epidermal growth factor receptor 2 positive (HER2+), four with estrogen receptor (ER) and progesterone receptor (PR) positive (ER+/PR+), 2 with triple negative (TNBC), and one with ER + positive (**Table S1**). The NSCLC subset of patients included 50% (*n* = 2) with an EGFR exon 19 mutation, 25% (*n* = 1) with EGFR amplification equivocal, and 25% (*n* = 1) with HER2-positive amplification. None of the patients had a known BRAF or ALK mutation. Eighty-seven percent of the patients were female with a median age of 59 years (Table 1). Twenty percent (*n* = 3) of patients had an Eastern Cooperative Oncology Group (ECOG) performance status of 0, 66.7% (*n* = 10) had an ECOG of 1, and 13.3% (*n* = 2) had an ECOG of 2.

**Table 1.** Patient Demographics and Baseline Disease Characteristics

Variable	*n*
**Age at study**	
*n*	15
Mean (STD)	57.9 (14.8)
Median (range)	59 (32, 82)
Interquartile	47, 69
**Gender**	
F (%)	13 (86.7)
M (%)	2 (13.3)
**Race**	
White (%)	15 (100)
**Ethnicity**	
Hispanic or Latino (%)	2 (13.3)
Non-Hispanic (%)	13 (86.7)
**Primary tumor site**	
Breast (%)	8 (53.3)
Lung (%)	4 (26.7)
Nasopharyngeal (%)	1 (6.7)
Ovary (%)	1 (6.7)
Pancreas (%)	1 (6.7)
**ECOG performance status scale**	
0 (%)	3 (20)
1 (%)	10 (66.7)
2 (%)	2 (13.3)
**Prior chemotherapy**	
Yes (%)	15 (100)
**Prior radiation therapy**	
No (%)	6 (40)
Yes (%)	9 (60
**Prior immunotherapy**	
No (%)	14 (93.3)
Yes (%)	1 (6.7)
**Prior surgery**	
No (%)	11 (73.3)
Yes (%)	4 (26.7)
**Prior hormonal therapy**	
No (%)	13 (86.7)
Yes (%)	2 (13.3)

Abbreviations: ECOG, Eastern Cooperative Oncology Group; F, female; M, ale; STD, standard deviation.

The median time between initial cancer diagnosis and study enrollment was 29 months (range, 6 to 200 months). All the patients had stable systemic, extracranial disease at enrollment and were heavily pretreated. All patients had received prior to enrollment systemic chemotherapy, 2 patients received prior hormonal therapy, 1 patient received prior immunotherapy, 9 patients received prior RT to a CNS site, and 4 had undergone prior surgery.

Patients were allowed to continue certain systemic therapies while on the study per PI discretion (see methods section). One patient was continued on Gefitinib; another patient on Herceptin and Perjeta; another patient on Osimertinib, carboplatin, pemetrexed, and bevacizumab; another patient received focal RT to the lumbro-sacral spine while on the study treatment; and another patient had a radioembolization to the liver lesion with 2 radiation seeds placed in the liver, and carboplatin while on the study treatment. Ten patients were not on any systemic treatment.

### Adverse Events

One of the primary endpoints was safety. DLTs were under the prespecified threshold following Pocock-type boundary; that is, the number of DLTs was below the prespecified threshold with full follow-up. Two patients developed DLTs within 28 days after the first dose of Avelumab. One of the patients developed adrenal insufficiency (grade 3) and hypothyroidism (grade 3), requiring hospitalization 28 days after the first dose of Avelumab. The second patient developed pneumonitis (grade 3) with acute hypoxemic respiratory failure at day 27 after receiving the first dose of Avelumab.

Grade 1 and 2 treatment-related AEs were observed in 5 (33%) patients, with 5 (33%) patients experiencing grade 3 or 4 toxicities, including hypoadrenalism, anemia, diarrhea, hypothyroidism, lymphopenia, thrombocytopenia, and leukopenia (**Table 2**). There were no grade 5 toxicities. The most frequently observed treatment-related AE was lymphopenia (grade 1–3). Immune-related AEs (ir-AEs) were seen in 6 (40%) patients (**Table 2**). Grade 1 or 2 ir-AEs occurred in 5 (33%) patients, including transaminitis, diarrhea, hypothyroidism, infusion site extravasation, lymphopenia, maculo-papular rash, and decreased white blood cell count. Grade 3 or 4 ir-AEs were detected in 3 (20%) patients, including hypoadrenalism (grade 3/4, *n* = 1), hypothyroidism (grade 3, *n* = 1), lymphopenia (grade 3, *n* = 1), and pneumonitis (grade 3, *n* = 1). There were no grade 5 ir-AEs.

**Table 2.** Adverse Events at Least Possibly Related to Treatment and Immune-Related Adverse Events (ir-AEs) After Treatment With Avelumab and Radiotherapy

Adverse events at least possibly related to treatment Avelumab and radiotherapy						
Toxicity	*N*	grade1 *n*	grade2 *n*	grade3 *n*	grade4 *n*	grade5 *n*
Adrenal insufficiency	15	-	-	1(6.7)	1(6.7)	-
Alanine aminotransferase increased	15	1(6.7)	1(6.7)	-	-	-
Alkaline phosphatase increased	15	1(6.7)	-	-	-	-
Anemia	15	2(13.3)	2(13.3)	1(6.7)	-	-
Anorexia	15	1(6.7)	1(6.7)	-	-	-
Arthralgia	15	1(6.7)	-	-	-	-
Aspartate aminotransferase increased	15	2(13.3)	-	-	-	-
Chills	15	1(6.7)	-	-	-	-
Constipation	15	1(6.7)	-	-	-	-
Diarrhea	15	2(13.3)	1(6.7)	1(6.7)	-	-
Eczema	15	-	1(6.7)	-	-	-
Fatigue	15	1(6.7)	2(13.3)	-	-	-
Fever	15	1(6.7)	-	-	-	-
Hypothyroidism	15	-	1(6.7)	1(6.7)	-	-
Infusion site extravasation	15	1(6.7)	-	-	-	-
Lymphocyte count decreased	15	4(26.7)	4(26.7)	3(20.0)	1(6.7)	-
Nausea	15	2(13.3)	2(13.3)	-	-	-
Neutrophil count decreased	15	1(6.7)	2(13.3)	-	-	-
Non-cardiac chest pain	15	1(6.7)	-	-	-	-
Platelet count decreased	15	2(13.3)	-	1(6.7)	-	-
Pneumonitis	15	-	-	1(6.7)	-	-
Rash maculo-papular	15	2(13.3)	-	-	-	-
Tremor	15	1(6.7)	-	-	-	-
Vomiting	15	2(13.3)	1(6.7)	-	-	-
White blood cell decreased	15	2(13.3)	1(6.7)	1(6.7)	-	-
Overall	15	1(6.7)	3(20.0)	4(26.7)	2(13.3)	0(0.0)
**Immune-related adverse events (ir-Aes) in patients treated with Avelumab and radiotherapy**						
**Toxicity**	***N***	**grade1 *n***	**grade2 *n***	**grade3 *n***	**grade4 *n***	**grade5 *n***
Adrenal insufficiency	15	-	-	1(6.7)	1(6.7)	-
Alanine aminotransferase increased	15	1(6.7)	1(6.7)	-	-	-
Alkaline phosphatase increased	15	1(6.7)	-	-	-	-
Aspartate aminotransferase increased	15	2(13.3)	-	-	-	-
Diarrhea	15	2(13.3)	1(6.7)	-	-	-
Hypothyroidism	15	-	1(6.7)	1(6.7)	-	-
Infusion site extravasation	15	1(6.7)	-	-	-	-
Lymphocyte count decreased	15	1(6.7)	1(6.7)	1(6.7)	-	-
Neutrophil count decreased	15	-	1(6.7)	-	-	-
Pneumonitis	15	-	-	1(6.7)	-	-
Rash maculo-papular	15	2(13.3)	-	-	-	-
White blood cells decreased	15	1(6.7)	-	-	-	-
Overall	15	1(6.7)	2(13.3)	2(13.3)	1(6.7)	0(0.0)

Toxicity attribution included “definite,” “possible,” and ‘probable.”.

All of the patients who had grades 3–4 AEs possibly, probably, and definitely related to Avelumab completely recovered from their toxicities, except for 2 patients who expired before follow-up assessment could be completed.

### Efficacy

**Figure 1.**
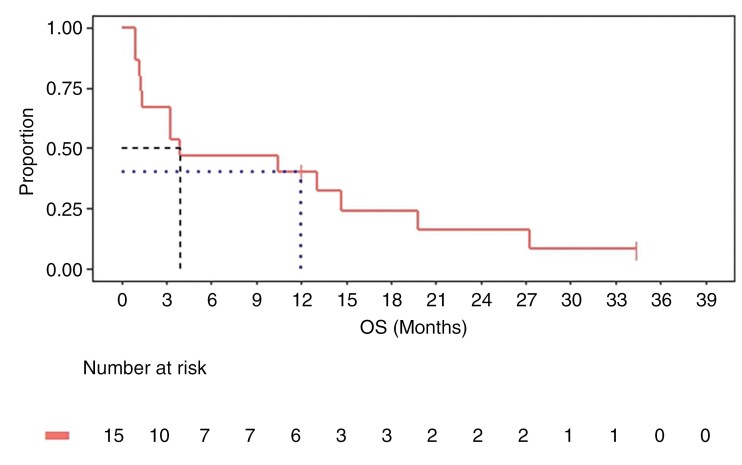
Overall survival (OS) after treatment with Avelumab and WBRT. Ten out of fifteen patients (66.7%; 95% CI: 38%–84%) were alive at 3 months after enrollment (OS3). Median OS was 3.85 months (95% CI: 0.9–34.4 months), and 1-year OS was 40% (95% CI: 16%–63%).

The proportion of patients alive at 3 months after the first dose of Avelumab (OS3) was one of the primary endpoints. The OS3 was 67% (10 of the 15; 95% CI: 38%–84%; **Figure 1**).

**Figure 2.**
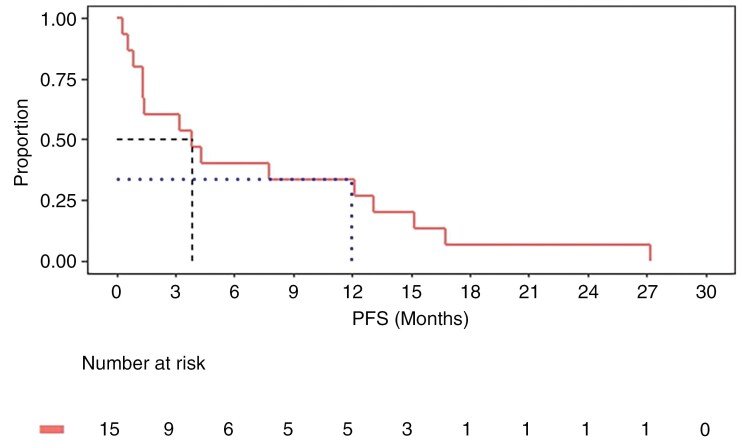
Progression-free survival (PFS) after treatment with Avelumab and WBRT. The median PFS was 3.85 months (95% CI: 0.9–12.1 months). At 12 months, PFS was 33% (95% CI: 12–56%).

OS and PFS were evaluated as secondary endpoints in the study. At a median follow-up of 3.7 months (range, 0.9–36.8 months; 95% CI: 1.3–14.7 months), the median OS was 3.85 months (95% CI: 0.9–34.4 months). The median follow-up time for survival analyses of the study was 19.8 months. The median PFS was 3.85 months (95% CI: 0.9–12.1 months; **Figure 2**). The OS rates at 3, 6, 9, 12, and 24 months were 67%, 47%, 47%, 40%, and 13.3%, respectively (**Figure 1** and **Table S2**). At 12 months, 6 of the 15 (40%) patients were alive (95% CI: 16%–63%; **Figure 1**), with a 12-month PFS of 33% (95% CI: 12%–56%). Two patients remained alive and clinically well after starting cycle 1, day 1 of treatment.

For the 8 patients with breast cancer, the OS3 rate was 62.5% (5 of the 8 patients; **Tables S1** and S**2**). The percentage of breast cancer patients alive at 9 and 12 months was 37.5% (3 of 8) and 25% (2 of the 8), respectively. The rate of hormone receptor (ER or PR positive) of patients alive at 3 months was 60% (3 of the 5) compared to 40% (2 of the 5) who did not survive to 3 months. Only one patient was HER2 + and had a survival of 34.42 months. The median OS for other subgroups was 5.07 months for HER2-, 4.81 months for ER + or PR+, 5.71 months for TNBC. For the 4 patients with NSCLC, the OS3 rate was 75% (3 of the 4 patients). The proportion of NSCLC patients alive at 12 months was 50% (2 of the 4). Two patients had extracranial disease progression and died from extracranial systemic disease complications. One patient developed complications from progressive ascites, and the other patient died from progressive systemic disease involving the bone marrow.

For CNS response, 4 of the 15 patients showed partial RR 0.27 (95% CI: 0.11, 0.52), and 5 of the 15 patients showed stable disease rate 0.33 (95% CI: 0.15, 0.58) as the best response. Four of the fifteen patients showed a progressive disease rate of 0.27 (95% CI: 0.11, 0.52), and 2 were not evaluable.

### Analysis of the Immune Environment of the CSF Samples From LMD Patients in the Study

**Figure 3.**
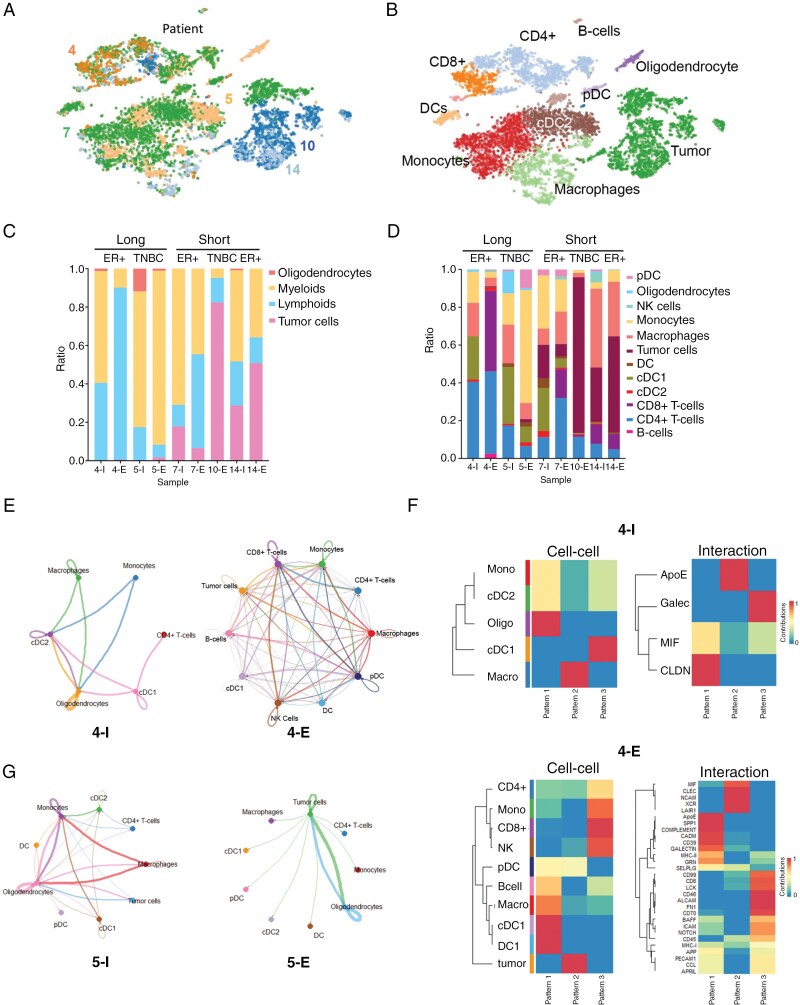
Defining the immune landscape of the CSF from patients treated with Avelumab + WBRT. (A) t-SNE plots showing cellular landscapes based on the sample of origin (patient numbers are indicated). (B) t-SNE plots showing cellular landscapes based on detailed cell typing. (I = initial sample, E = endpoint sample). (C) Proportion of cells from major cell types identified from each sample. Patient 4 = ER+/PR + breast cancer (BC) long-term responder; patient 5 = TNBC long-term responder; patients 7 and 14 = ER+/PR + BC short-term responder; patient 10 = TNBC short-term responder. (D) Detailed cell typing for each sample. (E), CellChat analysis showing the number of cell–cell interactions per cell type in samples 4I and 4E (= patient ER+/PR + BC long-term responder). (F) Identified patterns of cell–cell signaling and the genes associated with each signaling pattern for samples 4I and 4E through CellChat analysis. (G) CellChat analysis showing the number of cell–cell interactions per cell type in samples 5I and 5E (= patient TNBC long-term responder).

The immunocellular profiles of the CSF measured before and after treatment were part of the secondary endpoints of the study. We undertook scRNA-Seq analysis of CSF samples from a cohort of treated patients before treatment was initiated (samples labeled I for “Initial” sample) and then (where possible) at the end of the treatment cycle (samples labeled E for “Endpoint” sample). Nine samples of CSF were profiled, with 8 of these being patient-matched. The breakdown was 6 samples from 3 ER+/PR + breast cancer patients (1 long-term responder, 2 short-term responders: patients 4, 7, and 14) and 3 samples from 2 TNBC patients (1 long-term responder, 1 short-term responder: patients 5 and 10; **Figure 3A** and **Table S1**). Curation of the cell landscape identified lymphocytes (ie, CD4 + T cells, Tregs, CD8 + T cells, NK cells, B cells), myeloid cells (cDC1s, cDC2s, pDC, monocytes, and macrophages), tumor cells, and oligodendrocytes (**Figure 3A–D**). Individuals with tumor cells detectable in their CSF at baseline had a shorter survival (**Figure 3C and D**).

We used CellChat to infer the cell–cell interactions in the CSF at baseline and at the end of therapy.^36^ CellChat analysis allows the number of interactions and the strength of cell–cell communication to be inferred, and the shared patterns of signaling between the different cell types of the tumor microenvironment to be identified. Comparison of the initial and end-of-treatment samples demonstrated that Avelumab + WBRT was associated with an adaptive immune response, with increased CD4 + and CD8 + T cell and NK cell accumulation and interactions being noted (**Figure 3D–H**). Interestingly, this increase in CD8 + T-cell communication was even seen in patients with short survival (such as patients 7 and 10: **Figure S1**). An analysis of the baseline samples identified the main drivers of communication in the immune environment to be the oligodendrocytes, macrophages, and monocytes (samples 4I, 5I, 7I, and 14I: **Figure 3E–G** and **Figures S1****–S****2**). Comparison of the initial and endpoint samples identified 2 trends. The first, observed in samples 5I/5E and 14I/14E, was a loss of cell–cell communication in the endpoint sample, reflecting reduced activity in the immune compartment. The second was an increase in the number and strength of cell–cell interactions in the endpoint sample (observed in samples 4E and 7E; **Figure 3E–G** and **Figures S1****–S****2**). In this instance, the outgoing communication was driven largely by the macrophages, with ApoE, MIF, and SPP1 being identified as potential signals (**Figure 3F** and **Figure S2**). The potential importance of the macrophages in therapy failure was supported by the observation of strong outgoing communication from the macrophages to the other immune cell populations in 4/5 of the endpoint samples (4E, 7E, 10E, and 14E; **Figure 3E–G** and **Figures S1****–S****2**).

### LMD is Characterized by High Treg Activity at Baseline and T-cell Exhaustion at Progression

**Figure 4.**
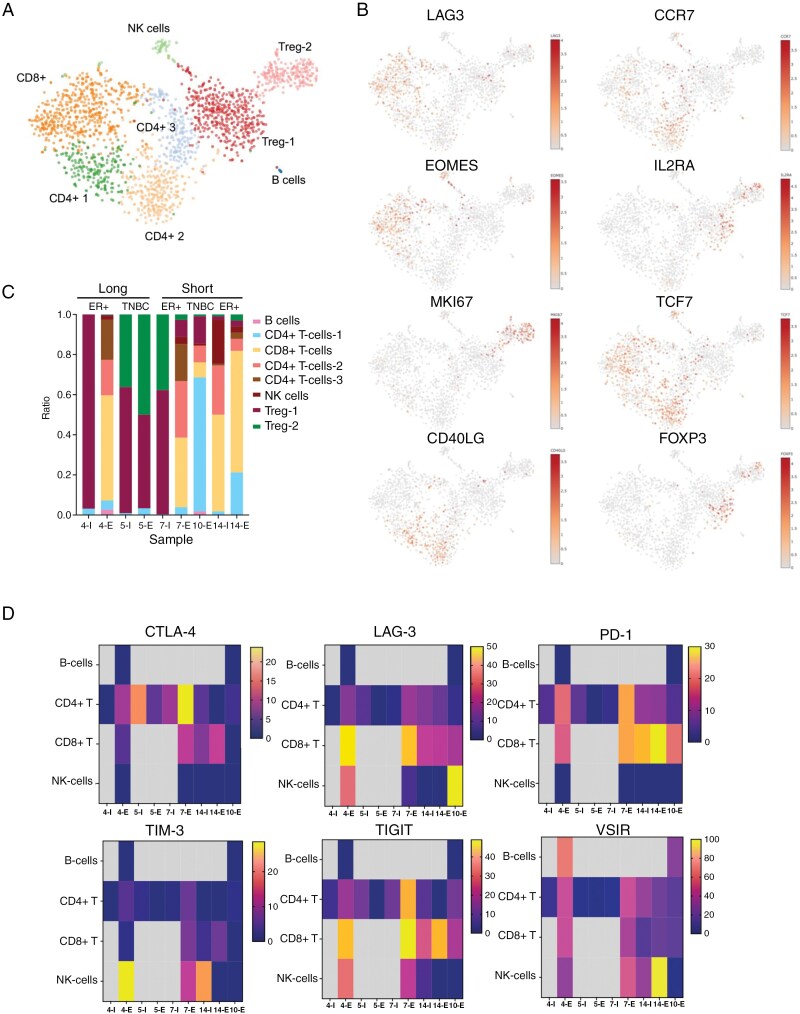
Analysis of the lymphocyte landscape of CSF specimens from patients treated with Avelumab + WBRT. (A) t-SNE plots showing the lymphocyte landscape of all samples analyzed. (B) t-SNE plots of key T-cell genes that allow the identified clusters to be differentiated. (C) Proportion of cells from major lymphocyte cell types identified from each sample (I = initial sample, E = endpoint sample). Patient 4 = ER+/PR + breast cancer (BC) long-term responder; patient 5 = TNBC long-term responder; patients 7 and 14 = ER+/PR + BC short-term responder; patient 10 = TNBC short-term responder. (D) Heatmaps show the expression of 6 immune checkpoints in the major subclasses of lymphocytes in each sample.

ICI therapy is predicated upon the reactivation of T-cell activity. We next addressed the effects of Avelumab + WBRT therapy on the lymphocyte repertoire of the CSF. Our cell-type curation identified 3 sub-clusters of CD4 + T cells, 2 clusters of regulatory T cells (Tregs), 1 cluster of CD8 + T cells, NK cells, and B cells (**Figure 4A,B and****Figure S3**). Only patients #4 (long-term responder ER+/PR + BC) and #10 (short-term responder TNBC) had evidence of B cells in their CSF at endpoint. Analysis of the T-cell compartment identified the CD8 + T cells to have high expression of LAG3, TCF7, and EOMES (**Figure 4B** and **Figure S3**). CD4 + T cell cluster 2 had high expression of CCR7 and CD40LG. The 2 Treg subclusters expressed high levels of FOXP3 and IL2RA, with Treg Cluster #2, being proliferative with high Ki67 (MKI67) positivity (**Figure 4B** and **Figure S3**).

One striking finding was the extremely high level of Tregs in some of the initial CSF samples, which accounted for >95% of the total lymphocyte population (samples 4I, 5I, and 7I; Figure 4C). Treatment with Avelumab + WBRT decreased the percentage of Tregs in some endpoint samples (4E and 7E: **Figure 4C**) and was associated with an increased accumulation of CD8 + T cells, CD4 + T cells clusters 2 and 3 (**Figure 4C**). An analysis of the lymphocyte gene expression profile demonstrated that although T-cell numbers increased at the end of treatment, these expressed markers of naïve T cells (CCR7, IL7R) as well as exhaustion and apoptotic/senescence markers (IFITM1, IL32; **Figure S4**).

We next analyzed the expression of clinically relevant immune checkpoints in the lymphocyte population. In the CD8 + T-cell compartment, PD-1 (PDCD1) and TIGIT were the most frequently expressed, with some patients, such as #4 and #7, exhibiting LAG3 expression at endpoint (**Figure 4D**). There was evidence of CTLA4, PD-1, TIGIT, and VSIR (Vista) being expressed in the CD4 + T cells. TIM3, LAG3, TIGIT, and VSIR were expressed in NK cells (**Figure 4D**). In the paired ER+/PR + breast cancer patients (eg, #4, 7, and 14), expression of CTLA-4, PD-1, TIGIT, and LAG3 were higher in the CD8 + T cells from end-of-treatment samples compared to the initial samples (**Figure 4D**). This did not seem to be related to survival.

## Discussion

The current study demonstrates that treatment with Avelumab + WBRT is safe and well-tolerated and has some activity in LMD that should be further evaluated in a Phase II trial. Advances in immunotherapy, including anti-PD-1, anti-CTLA-4, and anti-LAG-3 have led to improved survival in patients with metastatic cancers, including patients with brain metastases.^37–40^ As most of these studies excluded patients with LMD, the existing data for individuals with LMD are largely based on case reports.^41^ Only a handful of trials have specifically addressed the efficacy of immunotherapy in patients with LMD. These studies (summarized in **Table S3**) mostly focused on single-agent anti-PD-1 therapy, for example, pembrolizumab, nivolumab (IV), and nivolumab (IV + IT)^8,9,42^ and reported similar OS to our current study (OS3 of 66.7%: see **Table S3**).

While the effects of ICIs on the immune composition of CSF from LMD are not well understood, studies from our group and others have begun to characterize the CSF landscape with scRNA-seq. Analysis of the cell-type composition of CSF from patients with LMD is highly challenging. Numbers of cells in the CSF are usually very low (~100s to 1000s of cells per 7 ml), making standard immune profiling by flow cytometry very difficult (typically requiring 1–3 million cells). scRNA-Seq offers the advantage of very detailed cell curation and the identification of rare cell types, from very low cell numbers (10 000 cells or less). Some limitations of single-cell RNA profiling include difficulties in the detection of short-lived transcripts, such as interleukins.^43^ These studies showed the CSF from melanoma LMD is composed of a high number of apoptotic and exhausted CD4 + T-cells and a low number of CD8 + T-cells, suggesting an immune-suppressed microenvironment of exhausted T cells, as well having the presence of other myelosuppressive cells, including macrophages.^33^ The CSF obtained from a long-term survival patient with LMD who lived for more than 2 years after the LMD diagnosis showed an immune microenvironment similar to the CSF analyzed from patients without LMD.^33^ Further studies in CSF from patients with either breast or lung LMD exhibited similar immune-suppressed lymphocytic phenotypes.^44^ Given the rapidly expanding development of immunocellular therapies (eg, Bispecific T-cell engagers [BiTEs], CAR-T, dendritic cell vaccines, tumor infiltrating lymphocytes, it is essential to better understand the immunological landscape of the CSF of patients with LMD.

Albeit limited, the results from our scRNA-Seq analyses provided some insight into the effects of Avelumab + WBRT on the CSF immune landscape. As expected, the presence of tumor cells in the CSF at baseline, pretreatment (I = Initial), was associated with short survival. In-depth immune curation pretreatment identified CD4 + T cells, particularly Tregs, as the most predominant lymphocyte population at baseline. Of note, 2 distinct subtypes of Tregs were recognized, with one (Treg-2) expressing high levels of genes associated with proliferation, that is, Ki-67. It is likely that these different Treg populations have different effector functions, warranting further investigation in the context of LMD.^45^ In the ER + breast cancer, treatment with Avelumab + WBRT led to an increased accumulation of CD8 + T cells and memory-like CD4 + T cells and a decline in Tregs. There was also evidence for an increase in cell–cell interactions in some patients following treatment, demonstrating an increased adaptive immune response. As these were endpoint samples, the interactions were often macrophage-driven, hinting at a possible mechanism of immune escape. Our previous work on a series of CSF samples from the long-term survivor patient following treatment with anti-PD-1 demonstrated a potential link between increased macrophage numbers in the CSF and the onset of treatment resistance.^33^ In our current analysis, in the one long-term responder TNBC patient (patient #5), there was little evidence of CD8 + T-cell accumulation following treatment, but a decline in the proportion of Tregs. This is a complex picture of immune regulation with multiple potential mechanisms of response and resistance, and there is limited data from the few samples analyzed. Further studies with CSF interrogation would help elucidate light into the mechanisms behind Treg function in the pathophysiology of LMD.

All of the endpoint samples from the ER + breast cancer patients displayed an increase in the level of immune checkpoint expression (ie, CTLA-4, LAG3, TIGIT, and PD-1) in the CD8 + T cells. Of these, LAG3 showed the highest expression in CD8 + T cells, suggesting a novel therapeutic avenue to explore in LMD. There is already evidence that the combination of anti-PD-1 and anti-LAG3 is effective in mouse models of melanoma brain metastasis, warranting its future study in patients with LMD.^46^

Currently, multiple other clinical studies are underway in patients with LMD. These include a Phase 1 trial of a HER3-directed dendritic cell vaccine (NCT05809752), a Phase 2 study of ipilimumab and nivolumab (NCT02939300), a Phase 1 trial of intrathecal (IT) nivolumab (NCT05112549), a Phase 1 trial of Nivolumab and ipilimumab (both IT) in NSCLC and melanoma LMD (NCT05598853), a Phase 1/2B trial of nivolumab (IV + IT) in melanoma LMD (NCT03025256), a Phase 2 clinical trial of encorafenib, binimetinib, and nivolumab versus ipilimumab and nivolumab in BRAF^V600E^-mutant melanoma (NCT04511013), a Phase 2 trial of pembrolizumab (IV) and Lenvatinib (NCT04729348), a Phase 4 trial of durvalumab and methotrexate (IT) in NSCLC LMD (NCT04356222), and a Phase I trial of chimeric antigen receptor T-cell (CAR-T) therapy against HER2 in recurrent brain and leptomeningeal metastases (NCT03696030).

The antitumor activity of Avelumab is being studied in multiple tumor types; however, limited information is known in the setting of CNS metastases.^23–26^ Results from this study demonstrate that Avelumab can have some positive responses in the setting of other cancers (ie, breast, lung, nasopharyngeal, ovary, and pancreas cancers), with CNS metastases, opening avenues for further explorations in the preclinical and clinical research settings. Although the combination of anti-PD-L1 therapy and WBRT was well tolerated in our study, the use of WBRT has been superseded by pCSI or intensity-modulated RT (IMRT).^14^ Albeit limited, a few other centers have reported no difference in median OS between photon CSI and pCSI (1.7 vs 3 months, respectively),^11^ or modest OS of 4 months.^15^ Further prospective clinical trials will help clarify these discrepancies. At this time, the NCCN and the EANO-ESMO guidelines provide little information on the efficacy of pCSI in LMD. Since most institutions lack the technology to deliver pCSI, Avelumab might be appropriate to add to CSI IMRT, which will be included in future trials. It is also unknown how pCSI remodels the CSF immune microenvironment and its impact upon the recruitment and activation of peripheral immune cells. Since there are relatively few proton centers in the United States, we anticipate that most patients with LMD will continue to receive WBRT, and hence we regard our protocol as a reasonable approach to treat patients with LMD.

There are several limitations in the current study, including the small number of subjects enrolled and the focus on the assessment of safety and not efficacy. In addition, the population of patients we studied is heterogeneous, limiting generalizations for specific tumor and molecular subtypes. The heterogeneity of the population reflects the relative rarity of LMD, a common problem with designing and executing clinical trials for LMD patients. Furthermore, it is challenging to document CNS response in LMD, especially in small data sets.

Moreover, while CSF samples were acquired at different timepoints before and during treatment, lack of funding limited exploratory testing, which was completed on the CSF samples collected from breast cancer patients. We understand this weakens the translational component of the current study. Similarly, the immune landscape in the CSF correlated with the systemic immune response to treatment was not completed due to a lack of funding. The scRNA-Seq studies were limited not only by the small number of samples analyzed, but also by the inherent problems of studying RNA levels rather than cell surface levels of activation markers and secreted cytokines/chemokines. These technical limitations are a feature of CSF analysis, which typically contains very few cells (100–1000 cells per mL). A further limitation was the analysis of floating cell populations in the CSF rather than the cancer cells and associated immune cells growing on the surface of the leptomeninges. Autopsy specimens from cohorts of patients who succumb to LMD are difficult to obtain, and serial sampling of the leptomeninges is not possible. Further studies interrogating the CSF in correlation to systemic immune response would shed light on the pathophysiology of LMD.

## Summary

In summary, despite advances in systemic cancer therapy, LMD remains a devastating disease with a poor prognosis.^30^ This study shows that the combination of Avelumab and WBRT is safe, well-tolerated, with some activity in LMD. In this new era of immunotherapies, additional scientific studies and clinical trials need to be developed to explore the effects of ICIs, vaccines, and other immunotherapies alone or in combination with RT. The continued use of high-dimensional Omics approaches to interrogate the immune environment of the CSF (eg, cyto/chemokines, scRNA-Seq, genomics, etc.) will be essential to better understand the pathophysiology of LMD and patients’ response to treatment.

## Supplementary Material

noaf183_Supplementary_Figures_Tables_1

## Data Availability

All data and materials are stored in a safe, protected drive that is available for review.
