# Supplementary material for: Phase IB study of Avelumab and whole brain radiotherapy in patients with leptomeningeal disease from solid tumors: Results and molecular analyses
Source: Neuro Oncol. 2025 Aug 28;27(12):3237–49. doi: 10.1093/neuonc/noaf183 (PMC12916732; doi:10.1093/neuonc/noaf183)
Supplement: noaf183_Supplementary_Figures_Tables_1 [file noaf183_supplementary_figures_tables_1.docx]

**Supplementary Tables**

**Supplementary Table 1. Survival histological and molecular characteristics of patients treated with Avelumab and WBRT.**

| **Patient no.** | **Primary Cancer** | **Relevant Alterations** | **OS (months)** | **Long- vs. Short-Responders** |
| --- | --- | --- | --- | --- |
| **1** | NSCLC | EGFR exon 19 mut | 27.22 | long |
| **2** | breast | ER- PER- HER2+ | 34.42 | long |
| **3** | NSCLC | EGFR amp equivocal, FGFR1 amp equivocal, KRAS G12A | 19.82 | long |
| **4** | ***breast** | **ER+ PR+ HER2-** | **14.70** | **long** |
| **5** | ***breast** | **TNBC** | **10.49** | **long** |
| **7** | ***breast** | **ER+ PR+ HER2-** | **0.95** | **short** |
| **8** | NSCLC | HER2+ amp, ERBB2+ | 3.29 | short |
| **9** | NSCLC | EGFR exon 19 del mut | 1.32 | short |
| **10** | ***breast** | **TNBC** | **0.92** | **short** |
| **12** | undifferentiated sinus tumor | CD99-, CK 5/6 p63 INI-1 intact, eberish-, PDL1 3, +p16 | 13.08 | long |
| **13** | breast | ER 30%, PR-, HER2- | 3.22 | short |
| **14** | ***breast** | **ER+ PR+ HER2-, PIK3CA mut** | **1.35** | **short** |
| **15** | pancreatic adenocarcinoma | germline ATM, MLH1 RAD51D, intact MMR, mutant ERBB2 ie HER2, ROS1 | 1.18 | short |
| **16** | ovarian |  | 12.10 | long |
| **18** | breast | ER+ PR+ HER2- | 3.85 | short |
| *** Patients whose cerebrospinal fluid (CSF) was analyzed with single cell RNA-sequencing (scRNA-seq).** | | | | |

*Patients (4, 5, 7, 10, 14) whose cerebrospinal fluid (CSF) was analyzed with single cell RNA sequencing (scRNA-seq). Abbreviations: + = positive; - = negative; amp = amplification; EGFR = ; ER = estrogen receptor; HER2 = human epidermal growth factor receptor 2; mut = mutation; NSCLC = non-small cell lung cancer; OS = overall survival; PR = progesterone receptor; TNBC = triple negative breast cancer; WBRT = whole brain radiotherapy.

**Supplementary Table 2. Survival summary of patients treated with Avelumab and WBRT.**

| **Survival** | **n** | **%** |
| --- | --- | --- |
| Rate of OS at 3 months | | |
| Alive at 3 months | 10 | 67 |
| Not alive at 3 months | 5 | 33 |
| Rate of OS at 6 months | 7 | 47 |
| Rate of OS at 9 months | 7 | 47 |
| Rate of OS at 12 months | 6 | 40 |
| Rate of OS at 24 months | 2 | 13.3 |
| BC patients alive at 3 months | 5 | 62.5 |
| BC patients not alive at 3 months | 3 | 37.5 |
| BC patients alive at 9 months | 3 | 37.5 |
| BC patients not alive at 9 months | 5 | 62.5 |
| NSCLC alive at 3 months | 3 | 75% |
| NSCLC not alive at 3 months | 1 | 25% |

Abbreviations: BC = breast cancer; NSCLC = non-small cell lung cancer; OS = overall survival; WBRT = whole brain radiotherapy.

**Supplementary Table 3. Overall survival and progression free survival in several clinical trials in LMD using immunotherapy.**

| **Publication** | **Ph** | **Treatment** | **Tumor** | **N** | **3OS n(%)** | **mOS (mo)** | **6OS n(%)** | **12OS n(%)** |
| --- | --- | --- | --- | --- | --- | --- | --- | --- |
| Current study | 1 | Avelumab+WBRT | Breast, lung, other | 15 | 10 (67%) | 3.85 | 7 (47%) | 6 (40%) |
| Brastianos P.K. et al. Nat Med 2020 | 2 | Pembrolizumab | | 20 | 12 (60%) | 3.6 |  |  |
| Long G.V. et al. Lancet Oncol 2018 | 2 | Nivolumab IV | Melanoma | 16 | 1 (6%) patient had an intracranial response | | | |
| Glitza O. et al., Nat Med. 2023 | 1 | Nivolumab IV+IT | Melanoma | 25 | 17 (68%) | 4.9 | 11 (44%) | 26% |
| Ph = phase; OS = overall survival; 3OS = OS at 3 months; mOS = median OS; mo = months; 6OS = OS at 6 months; 12OS = OS at 12 months | | | | | | | | |

Abbreviations: 6OS = 6 months overall survival; 12OS = 12 months overall survival; IV = intravenous; IT = intrathecal; mOS = median overall survival; N = number of patients; OS = overall survival; Ph = phase; WBRT = whole brain radiotherapy.

**Supplementary Figures**


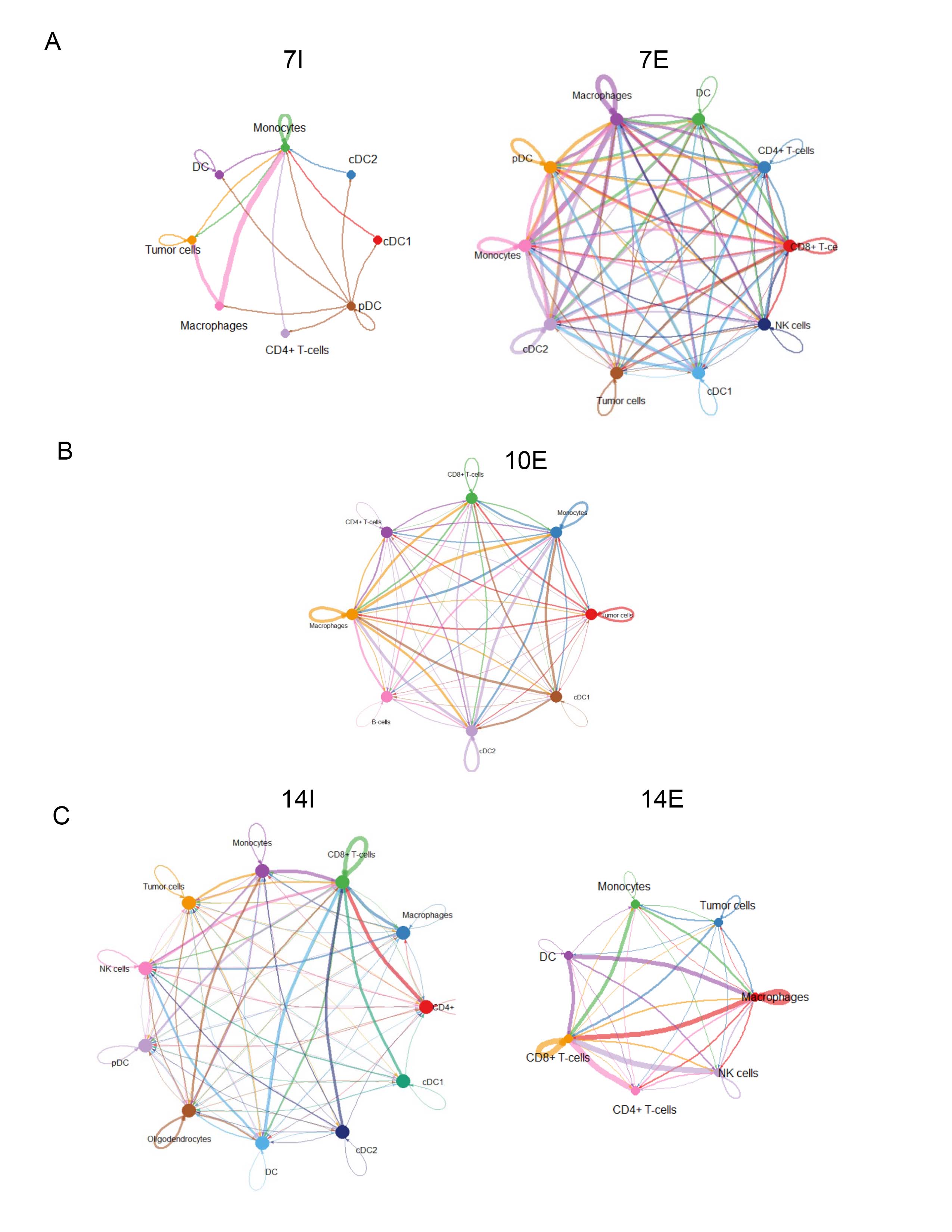


**Supplementary Figure 1:** Cell-cell interactions in CSF samples from patients with short responses. CellChat analysis was used to map the number of cell-cell interactions from patients 7, 10 and 14. I=initial sample, E=endpoint sample. Patient 4 = ER+/PR+ breast cancer (BC) long term responder; patient 5 = TNBC long term responder; patients 7 and 14 = ER+/PR+ BC short term responder; patient 10 = TNBC short term responder.


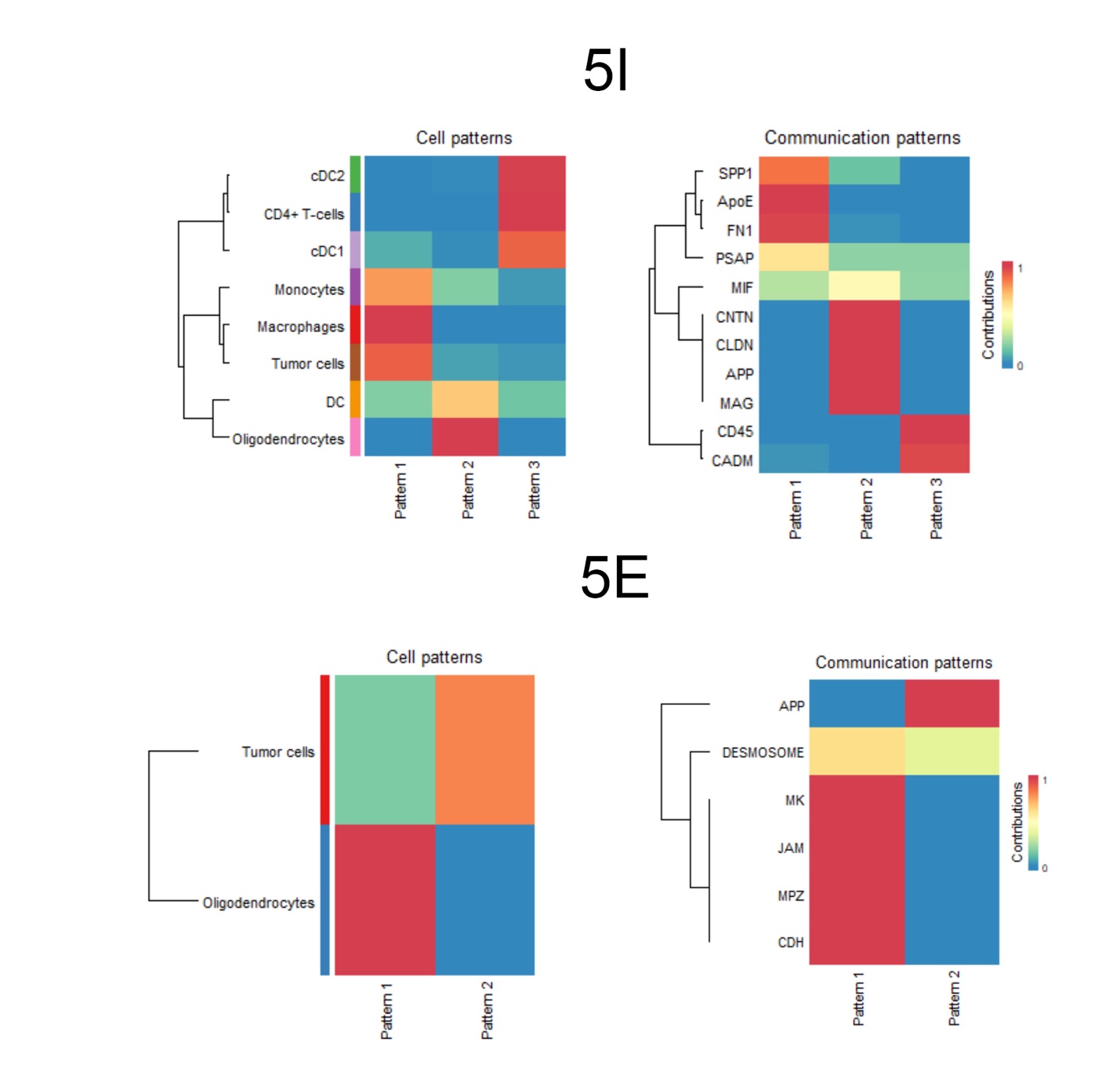


**Supplementary Figure 2:** Patterns of cell-cell interaction, along with associated genes for each pattern in long-term responder triple negative breast cancer (TNBC) patient 5. I=initial sample, E=endpoint sample.


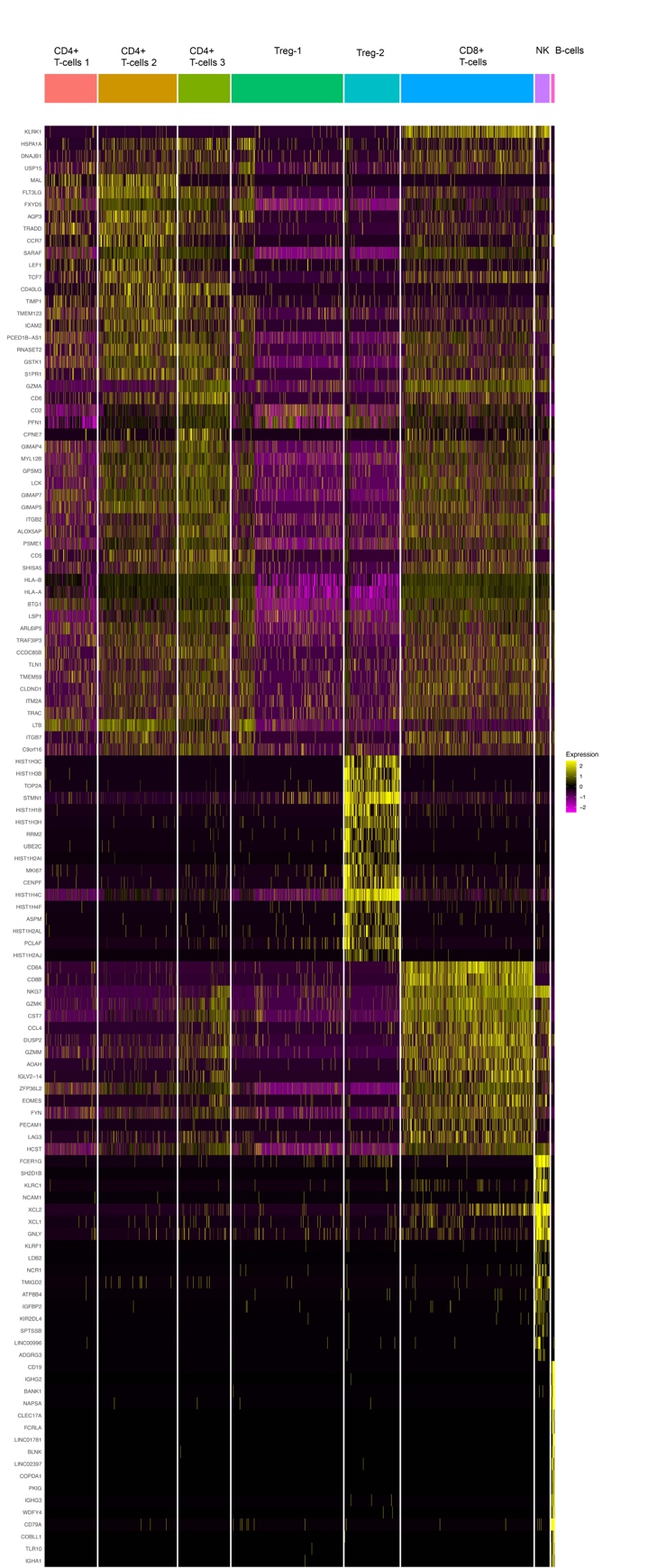


**Supplementary Figure 3:** Curation of the major lymphocyte subsets in the CSF samples. Heatmap shows the most differentially expressed genes between the identified subsets of CD4+, CD8+ T cells, Tregs, NK cells and B cells.


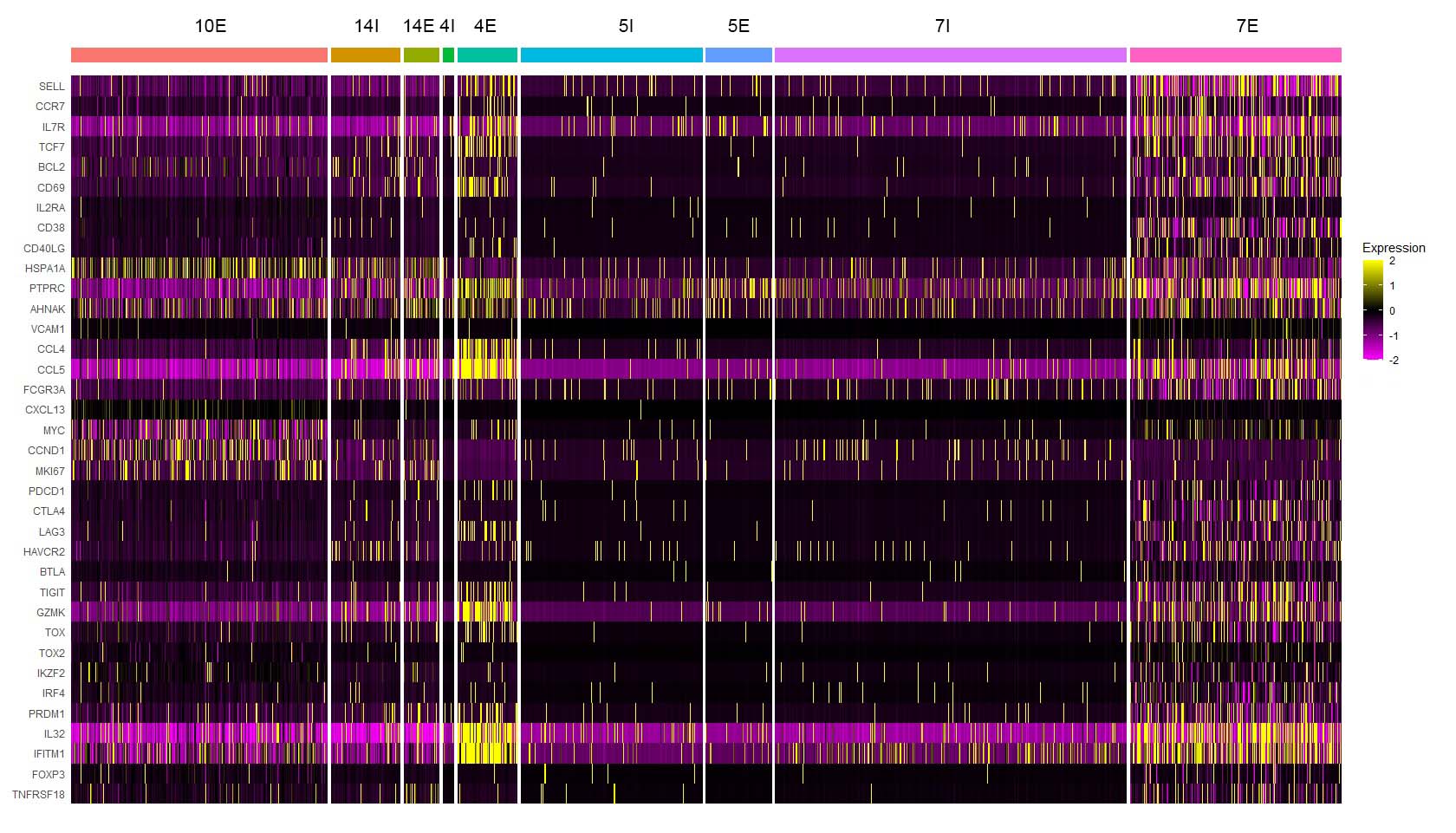


**Supplementary Figure 4:** Heatmap showing the expression of genes associated with naïve T cells, activation, exhaustion, immune checkpoints and apoptosis. Expression is shown per sample. I=initial sample, E=endpoint sample. Patient 4 = ER+/PR+ breast cancer (BC) long term responder; patient 5 = TNBC long term responder; patients 7 and 14 = ER+/PR+ BC short term responder; patient 10 = TNBC short term responder.
